# Nitinol Compression Staple Fixation Failure in Hand Surgery: A Report of Two Cases

**DOI:** 10.7759/cureus.87652

**Published:** 2025-07-10

**Authors:** Shigeki Ishibashi, Toko Umamoto, Maya Kanasaki, Reiko Tsukuura, Takumi Yamamoto

**Affiliations:** 1 Plastic and Reconstructive Surgery, National Center for Global Health and Medicine, Tokyo, JPN; 2 Orthopaedic Surgery, Graduate School of Biomedical and Health Sciences, Hiroshima University, Hirohsima, JPN

**Keywords:** cortical bone contact, fixation failure, nitinol compression staple, shape memory effect, short working length

## Abstract

Nitinol compression staples are increasingly used in hand surgery due to their shape memory effect and simplified surgical technique. While they have demonstrated favorable outcomes, reports on complications and failure mechanisms remain limited. We report two cases of fixation failure: one in a vascularized proximal interphalangeal joint transfer and another in thumb carpometacarpal joint arthrodesis. Both cases required revision surgery with plate fixation. These cases suggest that failure resulted from asymmetrical force distribution. This was caused by one staple leg achieving only monocortical purchase while the other was bicortical, combined with a short working length. These cases highlight the critical importance of preoperative planning and precise staple placement, particularly when bone dimensions are unequal or the working length is limited.

## Introduction

Nitinol (Ni-Ti alloy) compression staples are U-shaped implants that utilize the alloy's shape memory effect and superelasticity to provide sustained compressive force at bone fixation sites. Activated by body temperature, they maintain stable compression. The surgical procedure is also relatively simple, involving the creation of two drill holes parallel to the fracture line and subsequent insertion of the staple. Because of these features, nitinol compression staples have proven useful in the treatment of fractures of small and short bones, as well as in arthrodesis. Their clinical application has expanded in recent years, particularly in foot surgery [1-3].

This ability to generate sustained compression is rooted in the unique thermomechanical property of the alloy known as the shape memory effect. Nitinol exhibits two distinct solid-state phases: a low-temperature martensite phase and a high-temperature austenite phase. In its martensitic state, the material is highly flexible and can be easily deformed. However, when heated above its specific transformation temperature, it undergoes a phase transformation to the rigid austenite structure, forcefully reverting to its original, pre-set shape. For medical applications, this transformation temperature is engineered to be just below body temperature. Consequently, a staple, cooled and held in a deformed (open) position, will, upon insertion into the body and warming, attempt to return to its original (closed) shape. This process generates a constant, dynamic compressive force across bone fragments, facilitating stable fixation and promoting union.

In hand surgery, several successful outcomes have been reported for scaphoid fractures and intercarpal fusions [4,5]. For phalangeal and metacarpal fractures, Kirschner wire (K-wire) fixation and mini-plate/screw fixation remain the most common treatments [6-8]. However, in cases of simple transverse fractures, arthrodesis, or during bone grafting procedures for joint reconstruction, rapid compression fixation with nitinol staples may promote early bone union. Although limited, two clinical reports have demonstrated favorable postoperative outcomes in fracture treatment using these staples [9,10].

Bone fixation using nitinol compression staples has been reported to achieve results comparable to or better than those of conventional methods [1,5]. However, there are few detailed reports regarding complications and failures. We encountered two cases that resulted in fixation site failure. This report examines the potential causes of these failures and discusses preventative measures.

## Case presentation

Case 1

**Figure 1:**
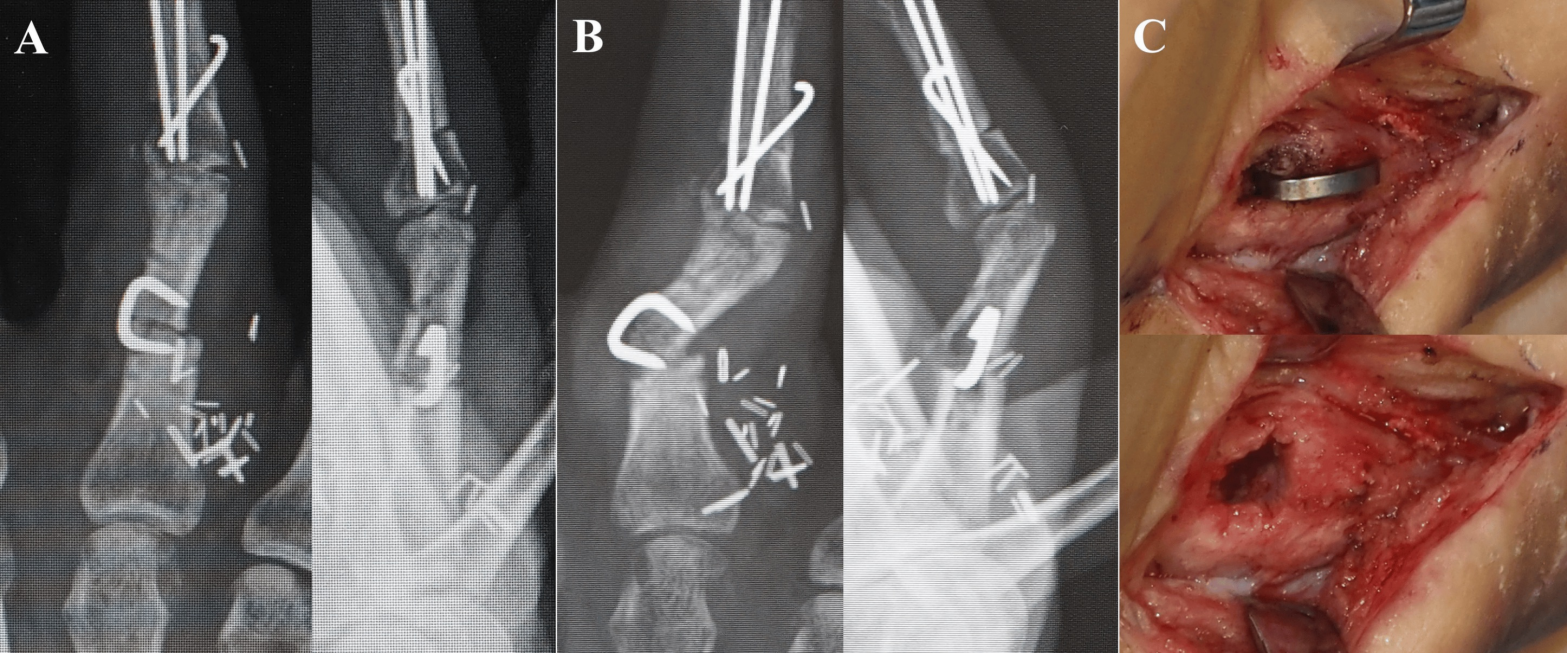
Use of a Nitinol compression staple for bone fixation in a proximal interphalangeal (PIP) joint transfer. (A) Post-fixation image. Intraoperative observation confirmed stable fixation with no gap at the host-graft junction. (B) Deformity of the finger was observed after initiating range-of-motion exercises. (C) Intraoperative findings during revision surgery, before and after staple removal, revealed bone destruction. The staple was easily removed.

A 32-year-old male sustained a right ring finger proximal interphalangeal (PIP) joint defect following a traffic accident. Joint reconstruction was performed using a vascularized joint transfer from the left second toe. Although initial bone fixation was attempted with Kirschner wires (K-wires), the proximal fixation site did not provide sufficient stability. Therefore, re-fixation was performed. Because the vascularized joint transfer required fixation in a limited surgical space while protecting the vascular pedicle and surrounding tissues, a nitinol compression staple (DynaNite, 9 mm x 7 mm, Arthrex, Inc., Naples, Florida, USA) was selected. After removing the K-wires, stable fixation was achieved with a staple (Figure 1A). Intraoperative observation confirmed stable fixation with no gap at the bone junction. After three weeks of external immobilization, the patient began active finger motion. The patient subsequently developed persistent pain, and radiographic examination revealed gap formation at the bone junction, suggesting fixation failure (Figure 1B). Revision surgery demonstrated bone destruction of the proximal phalanx (proximal segment), requiring plate fixation and autologous bone grafting (Figure 1C).

Case 2

**Figure 2:**
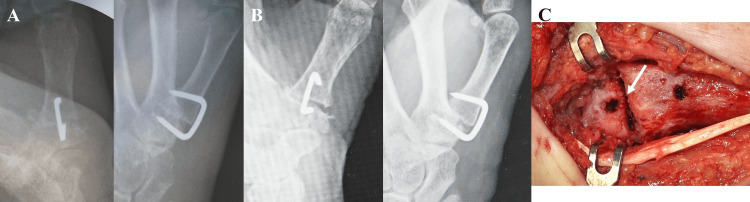
Use of a nitinol compression staple for arthrodesis of the thumb carpometacarpal (CMC) joint. (A) Intraoperative observation confirmed stable fixation with no gap at the fusion site. (B) Radiograph showing loosening at the fusion site after the patient reported pain following initiation of range-of-motion exercises. (C) Intraoperative photograph during revision surgery revealing a fracture around the staple leg (white arrow).

A 66-year-old female underwent arthrodesis for left thumb carpometacarpal (CMC) joint osteoarthritis. Following osteotomy of the first metacarpal and trapezium, fixation was performed using a Nitinol compression staple (DynaNite, 15 mm × 12 mm, Arthrex, Inc., Naples, Florida, USA) to utilize the sustained compression properties of the device. Intraoperative assessment showed satisfactory compression and stability at the fusion site (Figure 2A). Direct observation confirmed complete bone apposition with no gap at the bone junction. After three weeks of external immobilization, the patient began active motion exercises. She subsequently developed persistent pain, and radiographic evaluation revealed gap formation at the fusion site, suggesting staple loosening (Figure 2B). Revision surgery revealed a cortical bone fracture of the trapezium along with staple loosening, necessitating conversion to plate fixation (Figure 2C).

## Discussion

The primary biomechanical cause of failure in these two cases appears to be asymmetrical stress distribution resulting from unbalanced staple fixation. Specifically, this occurred when one staple leg achieved bicortical purchase while the other remained monocortical, leading to focal stress concentration on the cortex with a short working length, ultimately causing it to collapse. This report critically examines the technical and anatomical factors contributing to this asymmetrical fixation and proposes strategies to prevent such complications.

Factors contributing to staple fixation failure reported in the literature include patient-related factors such as underlying conditions and allergies, as well as biomechanical and technical factors. From a biomechanical perspective, the efficacy of Nitinol staples hinges on the limitations of its material properties, namely pseudoelasticity and the shape memory effect, which were explained in the introduction. The pseudoelasticity, which allows the material to absorb significant strain, assumes a uniform stress distribution [1,11]. In our cases, asymmetrical placement likely created intense local stress concentrations, particularly on the monocortical side, that may have surpassed this material threshold.

Furthermore, the temperature-dependent nature of the shape memory effect introduces another potential variable. It is conceivable that an imbalanced thermal environment between the two staple legs resulted in an uneven or incomplete austenite transformation. This thermal asymmetry would generate an unbalanced distribution of compressive strain across the fixation site, exacerbating the mechanical stresses already present from the staple's geometric placement. For example, in Case 1, the leg adjacent to the warm vascularized graft may have activated more rapidly or completely than the opposing leg. In Case 2, poor perfusion at the arthrodesis site may have created unpredictable thermal gradients, not only hindering the overall activation but also preventing the delivery of a uniform compressive force.

**Figure 3:**
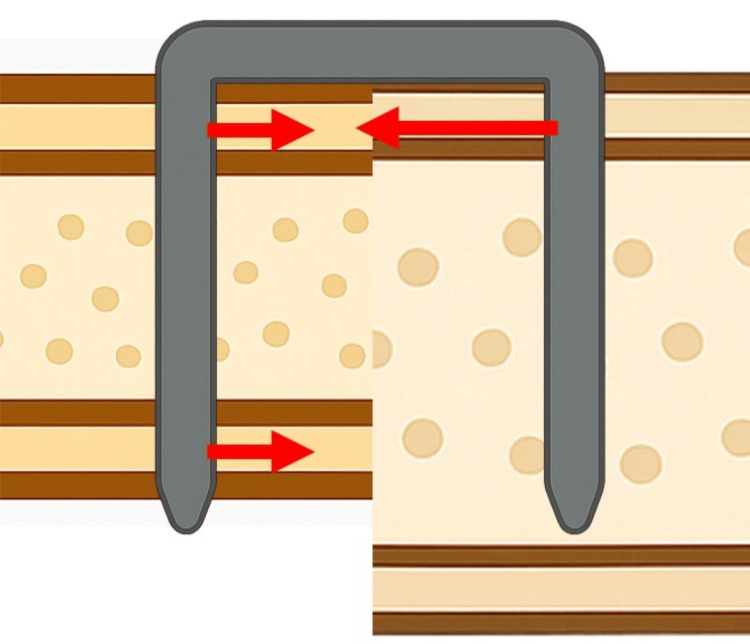
Schematic illustration of staple fixation failure in bones of unequal size. Asymmetrical fixation can occur when one staple leg achieves bicortical purchase while the other achieves only monocortical purchase. In this scenario, the compressive force on the monocortical side is highly concentrated, instead of being distributed across two cortices. This focal stress concentration can exceed the tolerance of the bone, leading to cortical fracture. The red arrows represent the vectors of compressive force; the arrow on the monocortical side is shown with a greater length to illustrate the larger magnitude of force concentrated on a single cortex. (Image created by author, S.I.)

The relatively thin profile of these staples, compared to other implants, makes them vulnerable to multi-axial loading forces. This can lead to breakage or loosening when used alone in unstable fractures or larger bones. In foot surgery, it has been noted that staples alone may provide insufficient fixation strength in cases with severe deformity or bone defects [12]. Experimental studies have suggested that excessively short staple legs may provide insufficient compression, and a single staple may fail to resist rotational or bending forces, potentially resulting in early failure. However, it has been reported that even when staple legs achieve only monocortical fixation, the strength is comparable to bicortical fixation if they reach within 2 mm of the cortex [13]. However, these tests were conducted with symmetrical placement of staple legs relative to the distal cortex. To date, there are no basic research studies examining the effects of asymmetrical staple placement. In the two cases reported here, differences in bone dimensions resulted in one staple leg failing to reach the distal cortex. This likely resulted in an asymmetrical distribution of compressive force, concentrating stress focally (Figure 3). Furthermore, the short working length of the cortex likely contributed to its collapse under this concentrated stress.

From a technical standpoint, precise staple insertion is critical for successful outcomes, and technical errors can lead to complications. First, drill holes must be created with appropriate depth and angle corresponding to the diameter and length of the staple legs. Insufficient pre-drilling risks cortical fracture during staple insertion. Additionally, interference between the staple bridge and tendons has been reported; therefore, near articular surfaces, the staple should be adequately countersunk to prevent protrusion [5]. Studies have demonstrated that countersinking does not compromise fixation strength [13].

In arthrodesis of the thumb CMC joint and bone fixation during PIP joint transplantation, the small dimensions of the bone fragments and epiphyses make precise staple leg placement critical. Unlike conventional screws, the required staple leg length cannot be measured intraoperatively due to the product specifications of nitinol compression staples. Some products have staples with different leg lengths on each side, highlighting the critical importance of detailed preoperative planning and careful intraoperative confirmation of accurate device placement. When fixation cannot be achieved as planned intraoperatively, when bone quality is poor with uncertain stability, or when symmetrical fixation is not obtained, consideration should be given to supplemental fixation methods, such as combining K-wire fixation or plate augmentation.

## Conclusions

Successful bone fixation with Nitinol compression staples hinges on achieving balanced, bilateral compression. Our cases illustrate that asymmetrical fixation can lead to early failure, even if initial stability appears adequate. Therefore, meticulous preoperative planning is essential, not only for choosing the appropriate staple size but also for assessing the feasibility of symmetrical placement. If symmetrical fixation is anatomically or technically challenging, surgeons should strongly consider alternative or supplemental fixation techniques to prevent complications. Further biomechanical studies, including finite element analysis, are warranted to validate the impact of asymmetrical loading on these implants.
